# Computational determination of human *PPARG* gene: SNPs and prediction of their effect on protein functions of diabetic patients

**DOI:** 10.1186/s40169-020-0258-1

**Published:** 2020-02-17

**Authors:** Howeida Abdullah Mustafa, Afraa Mohamed Suliman Albkrye, Buthiena Mohamed AbdAlla, Mona AbdelRahman Mohammed Khair, Nidal Abdelwahid, Hind Abdelaziz Elnasri

**Affiliations:** 1 Department of Molecular Biology and Bioinformatics, College of Veterinary Medicine, University of Bahri, Khartoum, Sudan; 2 Department of Biochemistry, Faculty of Veterinary Medicine, University of Khartoum, Khartoum, Sudan; 3 Department of Biochemistry, College of Applied and Industrial Science, University of Bahri, Bahri, Sudan

**Keywords:** Diabetes insilico, Polyphen, *PPARG*, SIFT, SNP

## Abstract

**Background:**

The Peroxisome proliferator-activated receptor gamma gene (*PPARG*), encodes a member of the peroxisome-activated receptor subfamily of nuclear receptors. *PPARs* form heterodimers with retinoid X receptors (RXRs) which regulate transcription of various genes. Three subtypes of *PPARs* are known: *PPAR*-*alpha*, *PPAR*-*delta* and *PPAR*-*gamma*. The protein encoded by this gene is *PPAR*-*gamma* which is a regulator of adipocyte differentiation. *PPARG*-*gamma* has been implicated in the pathology of numerous diseases including obesity, diabetes, atherosclerosis and cancer.

**Aim:**

This study aimed to perform insilico analysis to predict the effects that can be imposed by SNPs reported in *PPARG* gene.

**Methodology:**

This gene was investigated in NCBI database (http://www.ncbi.nlm.nih.gov/) during the year 2016 and the SNPs in coding region (exonal SNPs) that are non-synonymous (ns SNPs) were analyzed by computational softwares. SIFT, Polyphen, I-Mutant and PHD-SNP softwares). SIFT was used to filter the deleterious SNPs, Polyphen was used to determine the degree of pathogenicity, I-Mutant was used to determine the effect of mutation on protein stability while PHD-SNP software was used to investigate the effect of mutation on protein function. Furthermore, Structural and functional analysis of ns SNPs was also studied using Project HOPE software and modeling was conducted by Chimera.

**Results:**

A total of 34,035 SNPs from NCBI, were found, 21,235 of them were found in *Homo sapiens*, 134 in coding non synonymous (missense) and 89 were synonymous. Only SNPs present in coding regions were selected for analysis. Out of 12 deleterious SNPs sorted by SIFT, 10 were predicted by Polyphen to be probably damaging with PISC score = 1 and only two were benign. All these 10 double positive SNPs were disease related as predicted by PHD-SNPs and revealed decreased stability indicated by I-Mutant.

**Conclusion:**

Based on the findings of this study, it can be concluded that the deleterious ns SNPs (rs72551364 and rs121909244SNPs) of *PPARG* are important candidates for the cause of different types of human diseases including diabetes mellitus.

## Background

Type 2 diabetes is a complex disease characterized by elevated blood glucose, caused mainly by impairment in both insulin action and beta cell function. Although the sharp increase in prevalence of type 2 diabetes worldwide is attributed to changes in individual environmental exposure pattern, genetic factors may also predispose to the disease [1]. Type 2 diabetes mellitus (T2DM) is becoming increasingly prevalent throughout the whole world. The number of diabetic people is expected to increase from 387 million in 2014 to 592 million by 2035 according to the 6th Edition of the International Diabetes Federation’s (IDF) Diabetes Atlas [2]. The extensive application of genome-wide association studies (GWAS) in the identification of common genetic variants has greatly contributed to the discovery of diabetes susceptibility genes. Currently, at least 40 genetic loci have been convincingly associated with T2DM, including *KCNQ1*, *CDKAL1*, *TCF7L2*, *HMG20A*, *HNF4A*, *HNF1B*, and *DUSP9*. Several findings reported independent genome wide association (GWA) in Caucasians, which did not only confirm the effect of *PPARG*, *KCNJ11* and *TCF7L2*, but also identified six novel susceptibility loci including *CDKAL1*, *CDKN2A*-*CDKN2B*, *IDE*-*KIF11*-*HHEX*, *IGF2BP2*, *SLC30A8* and *FTO* [3–6]. The Peroxisome proliferator-activated receptor γ (*PPARγ*) is a nuclear hormone receptor preferentially expressed in adipose tissue. Activation by its ligand causes it to heterodimerize with the retinoid X receptor, bind specific DNA elements and induce a transcriptional cascade that leads to adipocyte differentiation and increased sensitivity to insulin [7]. The *PPARγ* molecule is now recognized as the cognate receptor for thiazolidinedione hypoglycaemic drugs [8].

According to Entrez-Gene, *PPAR* gamma gene maps to NC_000003 and spans a region of 100 kilo bases. According to Spidey, *PPAR* gamma 1 has 8 exons, the sizes being 171, 74, 228, 170, 139, 200, 451 and 459 bps. PPAR gamma 2 has 7 exons, the sizes being 173, 228, 170, 139, 200, 451 and 459 [9].

Single nucleotide polymorphisms (SNPs) are the most common genetic variations in any population; they occur when a single nucleotide in the genome (A, T, C or G) is altered [10]. They are present in every 200–300 bp in human genome [11]. So far, 5000,000 SNPs have been identified in the coding region of human population responsible for genetic variation diseases [12]. Among all SNPs, non-synonymous SNPs (ns SNPs) are present in exonic part of genome, which often leads to changes in amino acid residues of gene product. Even though many SNP’s have no effect on the biological functions of the cell, some can predispose people to certain diseases, influence their immunological response to drugs and can be considered as biomarkers for disease susceptibility [13]. Importantly, ns SNPs result in changes of the amino acid sequence of proteins and have been reported to be responsible for about 50% of all known genetic variations that are linked to inherited diseases [14]. On the other hand, coding synonymous (sSNPs) and those seen outside gene coding or promoter regions may also influence transcription factor binding and gene expression [15, 16].

Single nucleotide polymorphisms (SNPs) holds the key in defining the risk of an individual’s susceptibility to various illnesses and response to drugs. There is an ongoing process of identifying the common, biologically relevant SNPs, in particular those that are associated with the risk of disease. The identification and characterization of large numbers of these SNPs are necessary before we can begin to use them extensively as genetic tools [17].

### Justification

Diabetes mellitus is widely spreading within all ages. If uncontrolled it leads to very serious complications that would have very bad impact on diabetics and their families. *PPARG* was found to be a molecular target of insulin sensitizer hypoglycaemic drugs (Thiazolidinedione). Thus this study was carried out to predict the effect of *PPARG* SNPs on the function of the gene.

### Objectives

This study aimed to use Insilco analysis to predict the effects that can be imposed by SNPs reported in *PPARG*. The tools for fulfillment of the objective were a collection of computational softwares and databases including; NCBI-SNPs Database, GeneMania, Sorting Intolerant from Tolerant (SIFT), Polyphen, I-Mutant, PHD-SNPs, SNPs and Go, Project HOPE and Chimera.

#### Specific objectives

1. To obtain SNPs of PPARG gene from NCBI-SNPs Database.
2. To obtain *Homo sapiens* SNPs.
3. To analyze *Homo sapiens* SNPs for the deleterious ones [SIFT].
4. To analyze the degree of pathogenesity of SNPs [Polyphen].
5. To determine the effect of mutation on protein stability [I-Mutant].
6. To investigate the effect of mutation on protein structure [Project HOPE/Chimera].
7. To investigate the effect of mutation on protein function. [PHD-SNPs/Project HOPE].

## Materials and methods

### Data collection

Information regarding *PPARG* SNPs was obtained from National Center for Biological Information (NCBI) SNPs database in 2017. The SNPs and the related ensembles proteins (ESNP) were obtained from the SNPs database (dbSNPs) for computational analysis from http://www.ncbi.nlm.nih.gov/snp/ and Uniprot database [18]. The critical step in this study was to select SNPs for analysis by computational softwares. The selection was targeting SNPs in the coding region (exonal SNPs) that are non-synonymous (ns SNPs).

### GeneMania

GeneMania (http://www.genemania.org) is a web interface that helps predicting the function of genes and gene sets. GeneMania finds other genes that are related according to their function to the target study gene. The information provided by GeneMania include protein and genetic interactions between genes, pathways, co-expression, co-localization and protein domain similarity. GeneMania can be used to find new members of a pathway or complex and can also find additional genes which might have been missed in the screen. It can also find new genes with a specific function, such as protein kinases [19]. In this study the name of the gene was searched in the search window of the software and all the required information about the gene was obtained.

### Sorting intolerant from tolerant (SIFT)

SIFT (http://siftdna.org/www/SIFT_dbSNP.html) is an online software that predicts the tolerated and deleterious SNPs and detects the impact of amino acid substitution on protein function and phenotype alterations, so that users can list substitutions for further studies. The main principle of this program is that it generates alignments with a large number of homologous sequences and assigns scores to each residue ranging from 0 to 1. The threshold intolerance score for SNPs is 0.05 or less [20, 21]. In this study the SNPs rsIDs, were copied and pasted in the specified space within the software and the submit button was then clicked to obtain the result of sorting intolerant from tolerant SNPs. Then SNPs were copied in an excel sheet and they were filtered for the deleterious (intolerant) SNPs.

### Polymorphism phenotyping (polyphen-2)

Polyphen-2 (http://genetics.bwh.harvard.edu/pph2/) is an online bioinformatics softwares that automatically predict the effect of an amino acid change on the structure and consequently on the function of a protein. This prediction is based on the sequence and the effect of substitution on the structure and phylogeny. The mechanism of this program is based on multiple sequence alignment of 3D protein structure. It correlates information from different protein structure databases. Then it calculates the score of position-specific independent count (PSIC) for each variant. The higher the score, the greater is the effect of amino acid substitution. It identifies the prediction outcomes as benign (0–0.2), possibly damaging (0.2–0.85) and probably damaging (0.85–1).

In this study ns SNPs that were predicted to be intolerant by SIFT have been submitted to Polyphen as protein sequence in FASTA format obtained from Uniprot KB/Expasy after submitting the relevant ensemble protein (ESNP) there. The position of mutation was entered together with the native amino acid and the new substituents for both structural and functional predictions were noticed [22].

### I-Mutant

I-Mutant version 3.0 (http://gpcr2.biocomp.unibo.it/cgi/predictors/I-Mutant3.0/I-Mutant3.0.cgi) was used to predict protein stability changes in single-site mutations. I-Mutant basically can evaluate the stability change of a single site mutation starting from the protein structure or from the protein sequences [23]. In this study, the deleterious SNPs were submitted to I-Mutant server to predict protein stability changes in terms of support vector machine (svm2), predicted free energy change (DDG) and in terms of reliability index (RI).

### Predictor of human deleterious single nucleotide polymorphisms (PHD-SNP)

PHD-SNP is a web-based tool available at (http://snps.biofold.org/phd-snp/phd-snp.html2017). It predicts whether the new phenotype derived from a SNP is. Disease-related or not disease-related (neutral). In this study, the protein sequence obtained from Uniprot was submitted to the program after providing the position of mutation and the new amino acid residue [24].

### SNPs and Go

Is software that predicts the disease related mutations from protein FASTA sequence. Its output is prediction of results based on the determination among: disease related and neutral variations of protein sequence. The probability score higher than 0.5 reveals the disease related effect of mutation. (http://snps.biofold.org/snps-and-go//snps-and-go.html).

### Project HOPE

Project HOPE is web server that analyses the structural effects of intended mutation. HOPE co-operates with UniProt and DAS prediction servers in providing the mutated protein in an observable 3D structure. Data in Project HOPE, is entered in the form of protein sequence, then the mutant is selected and compared structurally with the wild type.

### Chimera

Chimera (http://www.cgl.ucsf.edu/chimera) is a high-quality extensible program for interactive conception and analysis of molecular assemblies and related data. This software is issued by University of California, San Francisco (UCSF). Chimera (version 1.8) was used to generate the mutated 3D model of each *PPARG* protein [25]. The PDB ID was fetched, preset and coloured. The sequence in the chain was presented, the region of mutation was selected and coloured. Atoms and bonds were exhibited and the structural model of the protein was obtained.

## Results and discussion

### Investigating the desired gene using dbSNPs/NCBI

*PPARG* gene was investigated in NCBI database (http://www.ncbi.nlm.nih.gov/). It contains a total of 34,035 SNPs, 21,235 of which are present in *Homo sapiens*, 134 were found in coding non synonymous regions (missense) and 89 were synonymous.

### GeneMania

**Fig. 1:**
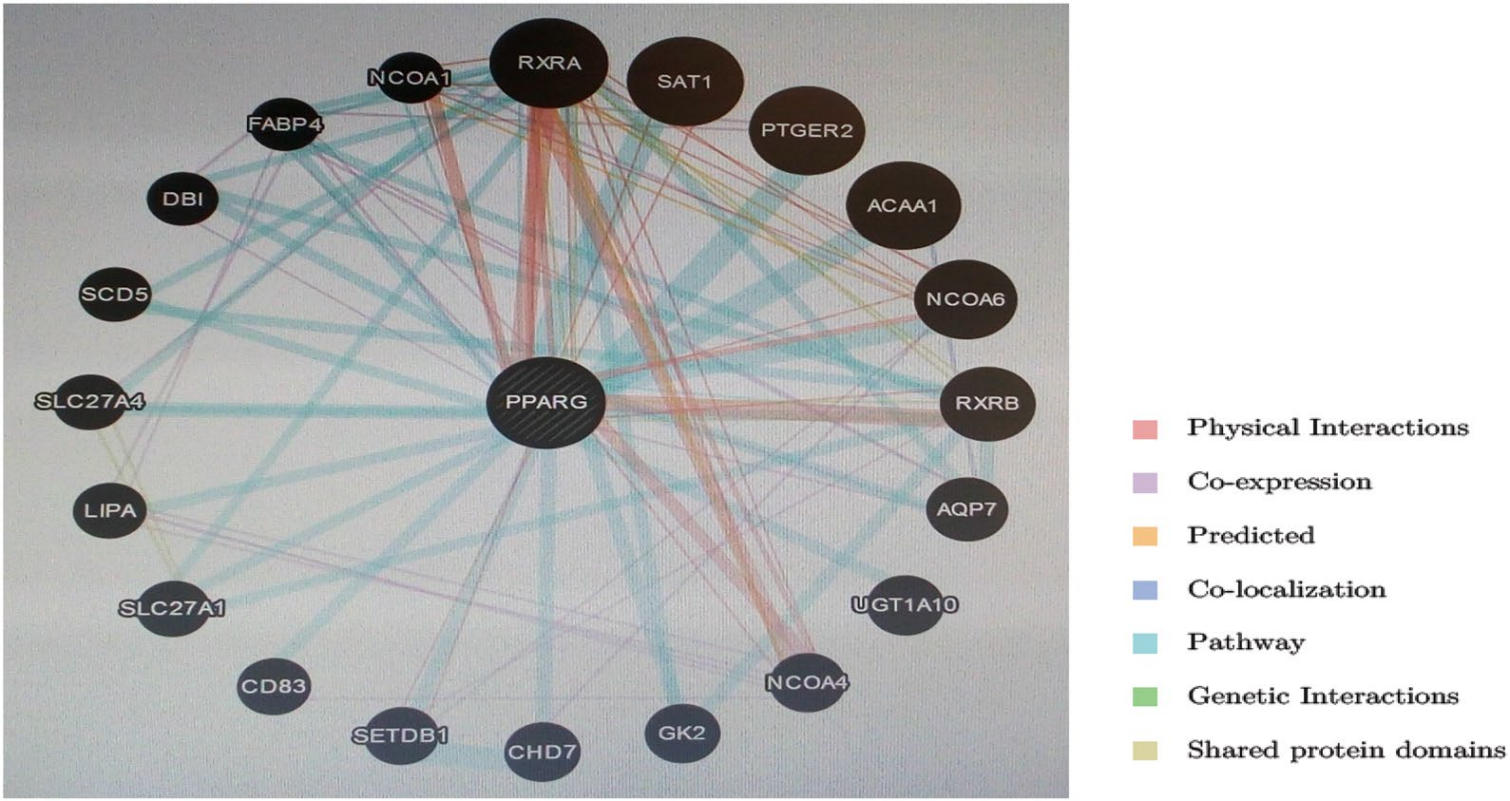
Genes cogene-expressed with *PPARG* gene

*PPARG* plays an important role in nuclear hormone receptor binding, hormone receptor binding, intracellular receptor signaling pathway, long chain fatty acid transport and transcription initiation from RNA Polymerase II Promotor *PPARG* gene has a vital role in human body. The findings revealed that *PPARG* is co-expressed with 4 genes (*RXRA*, *RXRB*, *AQP7* and *FABP4*) and shared domain with only 2 genes (*RXRA* and *RXRB*) as listed in Fig. 1 and Table 1.

**Table 1:** Genes co-expressed and sharing a domain with PPARG

Gene Symbol	Description	Co-expression	Shared domain
RXRA	Retinoid X receptor alpha	Yes	Yes
SAT1	Spermidine/spermine N1-acetyltransferase 1	No	No
PTGER2	Prostaglandin E receptor 2	No	No
ACAA1	Acetyl-CoA acyltransferase 1	No	No
NCOA6	Nuclear receptor coactivator 6	No	No
RXRB	Retinoid X receptor beta	Yes	Yes
AQP7	Aquaporin 7	Yes	No
UGT1A10	UDP Glucuronosyltransferase family1 member	No	No
NCOA4	Nuclear receptor coactivator 4	No	No
GK2	Glycerol kinase 2	No	No
CHD7	Chromodomain helicase DNA binding protein 7	No	No
SETDB1	SET Domain bifurcated 1	No	No
CD83	CD83 Molecule	No	No
SLC27A1	Solute carrier family 27 member 1	No	No
LIPA	Lipase A, lysosomal acid type	No	No
SLC27A4	Solute carrier family 27 member 4	No	No
SCD5	Stearoyl-CoA desaturase 5	No	No
DBI	Diazepam binding inhibitor, acyl-CoA binding protein	No	No
FABP4	Fatty acid binding protein 4	Yes	No
NCOA1	Nuclear receptor coactivator 1	No	No

### Prediction of SNPs in coding region

Non synonymous SNPs were analyzed by SIFT software. Out of 12 SNPs (according to their related ensemble proteins), 10 were predicted to be deleterious (Table 2). They were also found to be probably damaging using Polyphen with a high score (= 1) (Table 3). In another study [25], which dealt with type 2 diabetes mellitus (T2D) drug responsiveness associated SNPs, analysis of SNP ID (rs1801282) of gene *PPARG* showed a single positive effect by SIFT (deleterious) while Polyphen analysis revealed that it is benign. In this current study, this is similar to SNP IDs (rs72551364 and rs121909244) in being deleterious by SIFT and benign by Polyphen.

**Table 2:** *PPARG* functions and its appearance in network and genome

Function	FDR	Coverage	
		Genes in network	Genes in genome
Nuclear hormone receptor binding	6.81 E–4	5	94
Hormone receptor binding	6.81 E–4	5	103
Transcription co-activator activity	1.20 E–3	6	249
Intracellular receptor signaling pathway	1.09 E–2	5	207
Long chain fatty acid transport	2.03 E–2	3	33
Ligand activated sequence-specific DNA binding RNA polymerase II transcription factor activity	2.03 E–2	3	44
Direct ligand regulated sequence-specific DNA binding transcription factor activity	2.08 E–2	3	36
Ligand-dependent nuclear receptor transcription co-activator activity	2.51 E–2	3	40
Fatty acid transport	3.19 E–2	3	45
Steroid hormone receptor binding	6.85 E–2	3	60
Transcription initiation from RNA Polymerase II Promotor	8.00 E–2	4	190
Peroxisome proliferator activated receptor signaling pathway	8.00 E–2	2	10

**Table 3:** Nonsynonymous SNPs predicted with SIFT, Polyphen, I-Mutant and PHD-SNP programs, chosen SNPs with PSIC SD range (1–0.99) and Tolerance Index equal (0.009)

SNP ID	Protein ID	Amino acid change	SIFT		Polyphen		I Mutant		
			SIFT prediction	SIFT score	Polyphen prediction	Polyphen score	SVM2 prediction effect	DDG	RI
rs72551364	ENSP00000287820	R425C	Deleterious	0	Probably damaging	1	Decrease	− 2.38	7
rs72551364	ENSP00000312472	R397C	Deleterious	0	Probably damaging	1	Decrease	− 0.67	7
rs72551364	ENSP00000380205	R397C	Deleterious	0	Probably damaging.	1	Decrease	− 0.67	7
rs72551364	ENSP00000380207	R397C	Deleterious	0	Probably damaging	1	Decrease	− 0.67	7
rs72551364	ENSP00000380210	R397C	Deleterious	0	Probably damaging	1	Decrease	− 0.67	7
rs72551364	ENSP00000380221	R403C	Deleterious	0	Benign	0.356	Decrease	− 0.85	7
rs121909244	ENSP00000287820	P495L	Deleterious	0.001	Benign	0.356	Decrease	− 0.19	4
rs121909244	ENSP00000312472	P467L	Deleterious	0.001	Probably damaging	1	Decrease	− 0.14	4
rs121909244	ENSP00000380205	P467L	Deleterious	0.001	Probably damaging	1	Decrease	− 0.14	4
rs121909244	ENSP00000380207	P467L	Deleterious	0.001	Probably damaging	1	Decrease	− 0.14	4
rs121909244	ENSP00000380210	P467L	Deleterious	0.001	Probably damaging	1	Decrease	− 0.14	4
rs121909244	ENSP00000380221	P473L	Deleterious	0.001	Probably damaging	1	Decrease	0.46	4

### Prediction of change in stability due to mutation using I-Mutant 3.0 server

All the 10 nonsynonymous SNPs (according to their related ensemble proteins) that were predicted to be deleterious and damaging by both SIFT and Polyphen softwares (double positive), were submitted to the I-Mutant 3.0 server. The outcomes predicted that all the mutations in *PPARG* gene revealed decreased protein stability as illustrated in Table 3.

### Association of ns SNPs to disease using PHD-SNP and determination of probability score using SNPs and Go softwares

All the 10 nonsynonymous SNPs (according to their related ensemble proteins) that were predicted to be deleterious and damaging by both SIFT and Polyphen softwares were submitted to the PHD-SNP and then to SNPs and Go softwares. The findings revealed that all of them were predicted to be disease related with RI equals 5 and 6 as demonstrated in (Table 4).

**Table 4:** Nonsynonymous SNPs predicted with PHD-SNPs & SNPs & Go programs, chosen SNPs with PSIC SD range (1–099) and tolerance index equal (0.009)

SNP ID	Protein ID	Amino acid change	PHD-SNPs		SNPs & Go	
			PHD-SNP effect	RI	SNPs & Go prediction	RI
rs72551364	ENSP00000287820	R425C	Disease	7	Disease	6
rs72551364	ENSP00000312472	R397C	Disease	7	Disease	6
rs72551364	ENSP00000380205	R397C	Disease	7	Disease	6
rs72551364	ENSP00000380207	R397C	Disease	7	Disease	6
rs72551364	ENSP00000380210	R397C	Disease	7	Disease	6
rs72551364	ENSP00000380221	R403C	Disease	7	Disease	6
rs121909244	ENSP00000287820	P495L	Disease	4	Disease	5
rs121909244	ENSP00000312472	P467L	Disease	4	Disease	5
rs121909244	ENSP00000380205	P467L	Disease	4	Disease	5
rs121909244	ENSP00000380207	P467L	Disease	4	Disease	5
rs121909244	ENSP00000380210	P467L	Disease	4	Disease	5
rs121909244	ENSP00000380221	P473L	Disease	4	Disease	5

### Findings of project HOPE software

All the 10 non synonymous SNPs that were predicted to be deleterious and damaging by both SIFT and Polyphen softwares were submitted to Project HOPE software. The findings revealed that rs72551364 resulted in substitution of Arginine (wild type) to Cysteine (mutant) at positions (425, 397 and 403). The mutant residue (Cysteine) is smaller than the wild-type residue Arginine which is positively charged while the mutant (Cysteine) is neutral. Arginine is more hydrophobic than Cysteine. The size difference between wild-type (Arginine) and mutant residue (Cysteine), results in an inaccurate position for the new residue to make the same hydrogen bond as the original wild-type residue. The difference in hydrophobicity affects hydrogen bond formation. The wild-type residue (Arginine) forms a salt bridge with: (Glutamic Acid at position 330) and (Aspartic Acid at position 402).The difference in charge leads to disturbance of the ionic interaction made by the original, wild-type residue (Arginine). The differences in amino acid properties can disturb this region and disturb its function, according to Project HOPE. Its pathogenicity can be attributed to loss of its hypophobicity (as detected by PHD-SNPs software) and also related to the decreased stability (as predicted by I-Mutant software) [26].

The rs121909244 resulted in substitution of a Proline (wild type) to Leucine (mutant) at positions (467 and 473). The mutant residue (Leucine) is bigger than the wild-type residue (Proline). Prolines are known to be very rigid and therefore induce a special backbone conformation which might be required at this position. The mutation can disturb this special conformation. The mutant residue (Leucine) is bigger than the wild-type residue (Proline) which is located on the surface of the protein, mutation of this residue can disturb interactions with other molecules or other parts of the protein, according to Project HOPE. Reduced rigidity of the mutant (Leucine) is predicted to be disease related by PHD-SNPs software and to decrease effective stability of protein using I mutant software. This confirms the pathogenicity of the SNP.

### Chimera

**Fig. 2:**
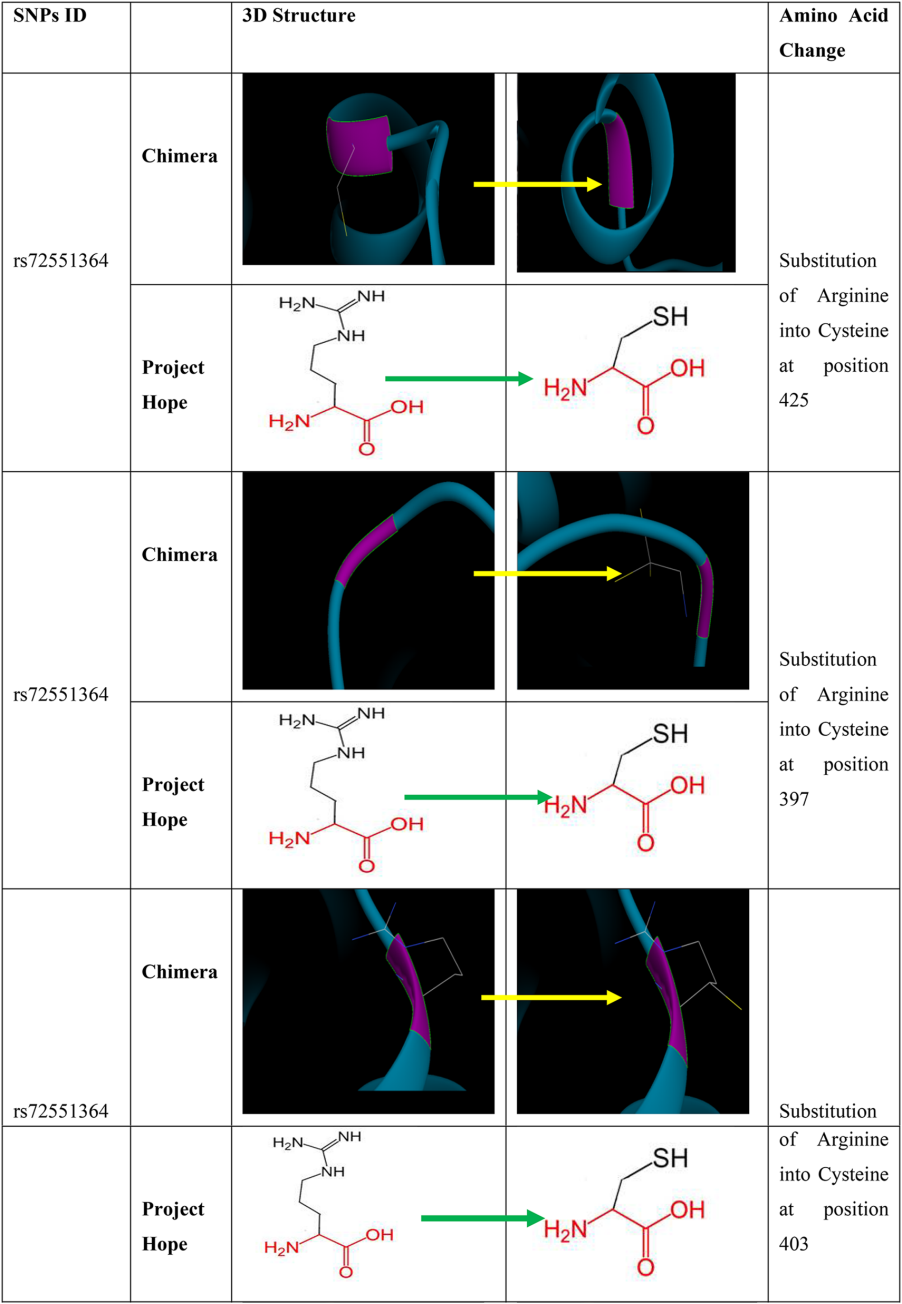

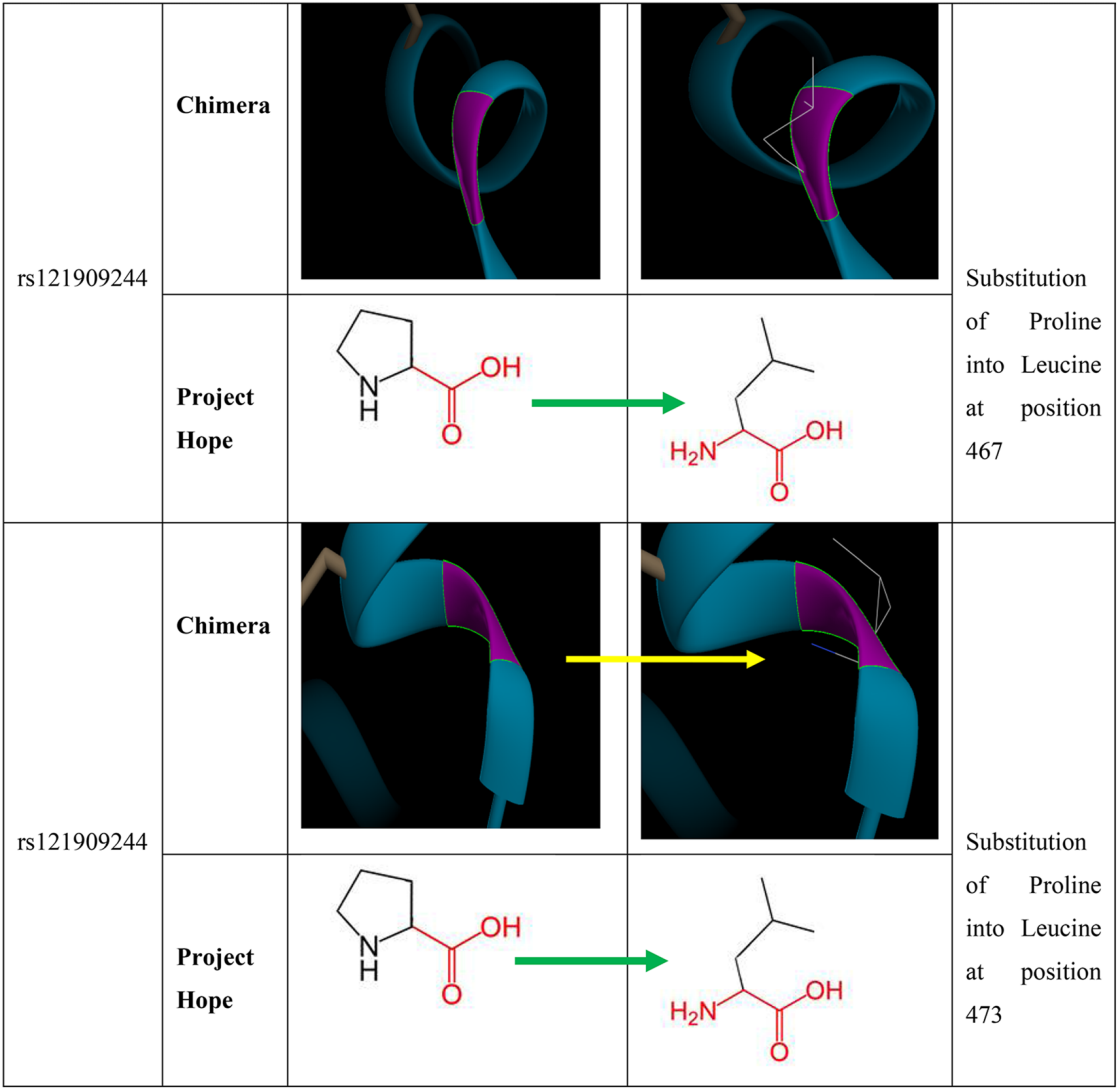
3D model by Chimera and Project HOPE for PPARG protein

Chimera program has been used to visualize the PDB file of rs72551364 and rs121909244SNPs and to determine the position of the mutant and replace it with the new amino acid (Fig. 2).

Peroxisome proliferator-activated receptor-gamma (*PPAR*-*γ*) is a transcription factor that plays a vital role in activation of adipocyte differentiation and is an important modulator of gene expression in a number of specialized cell types, including adipocytes, where it acts by regulating the transcription of numerous target genes [27]. The primary effect of *PPARG* seems to be on body weight; at least 10 studies have shown an association between the ALA allele and higher Body Mass Index (BMI) or obesity [23]. Human *PPAR*-*γ* expression was first described in hematopoietic cells and later also in spleen, liver, testis, skeletal muscle, and brain, in addition to fat [28]. (*PPAR*-*γ*) signaling pathways affect both cellular and systemic lipid metabolism and have links to obesity, diabetes and cardiovascular disease [29]. The ALA allele was shown to have reduced efficiency in trans-activating responsive promoters [30] and a reduced ability to stimulate adipogenesis in response to activation of thiazolidinedione [31]. Nonetheless, results of studies on the association with this variant in man have been variable, both regarding the ability to detect an effect on obesity or glucose homeostasis and the direction of such effect [32–34].

10 SNPs were predicted by this current study to be the most damaging or disease related SNPs in *PPARG* Gene. It can be proposed that these 10 most deleterious SNPs of *PPARG* gene may be involved in the pathogenesis of the *PPARG*-associated diseases as mentioned in the above studies. This can be attributed to the association of these diseases.

## Conclusion

Functional and structural impact of SNPs in the *PPARG* gene was studied using computational prediction tools. Out of the total of 21,235 *Homo sapiens*, 134 in coding non synonymous (missense) and 89 synonymous. In order to make effective use of genetic diagnosis, the predicted harmful SNPs in the *PPARG* gene are recommended to be well known and available to the diagnostic services and molecular biology laboratories to ensure accurate diagnosis for the associated diseases which can also lead to successful intervention. Based on this study, it is predicted that (rs72551364 and rs121909244SNPs) are important candidates for the cause of different types of human diseases caused by *PPARG* gene.

## Data Availability

All data analyzed during the study are included in the article.

## Abbreviations

SNPs: Single nucleotide polymorphisms
*PPARG*: the peroxisome proliferator-activated receptor gamma gene
RXRs: retinoid X receptors
NCBI: National Centre for Biotechnology Information
ns SNPs: non-synonymous
SIFT: sorting intolerant from tolerant
Polyphen-2: polymorphism phenotyping-2
PHD-SNP: predictor of human deleterious single nucleotide polymorphisms
UCSF: University of California, San Francisco
T2DM: type 2 diabetes mellitus
IDF: International Diabetes Federation
GWAS: genome-wide association studies

## References

[1] Florez JC, Jablonski KA, Sun MW, Bayley N, Kahn SE, Shamoon H (2007). Effects of the type 2 diabetes-associated *PPARG* P12A polymorphism on progression to diabetes and response to troglitazone. J Clin Endocrinol Metab..

[2] Fernandes J, Ogurtsova K, Linnenkampa U, Guariguata L, Seuringa T, Zhang P, Cavana D, Makaroff LE (2016). IDF Diabetes Atlas estimates of 2014 global health expenditures on diabetes. Diabet res Clin Pract..

[3] Zhang W, Wang H, Guan X, Niu Q, Li W (2015). Variant rs2237892 of KCNQ1 is potentially associated with hypertension and macrovascular complications in type 2 diabetes mellitus in a Chinese Han population. Genomics Proteomics Bioinform..

[4] Zeggini E, Weedon MN, Lindgren CM, Frayling TM, Elliott KS (2007). Replication of genome-wide association signals in UK samples reveals risk loci for type 2 diabetes. Science.

[5] Saxena R, Voight BF, Lyssenko V, Burtt NP, de Bakker PI (2007). Genome-wide association analysis identifies loci for type 2 diabetes and triglyceride levels. Science.

[6] Scott LJ, Mohlke KL, Bonnycastle LL, Willer CJ, Li Y (2007). A genome wide association study of type 2 diabetes in Finns detects multiple susceptibility variants. Science.

[7] Sladek R, Rocheleau G, Rung J, Dina C, Shen L (2007). A genome-wide association study identifies novel risk loci for type 2 diabetes. Nature.

[8] Spiegelman BM (1998). PPAR-γ: adipogenic regulator and thiazolidinedione receptor. Diabetes.

[9] http://atlasgeneticsoncology.org/Gene/PPARGID383ch3p25.html. Accessed 19 Oct 2019

[10] Lehmann JM, Moore LB, Smith-Oliver TA, Wilkison WO, Willson TM, Kliewer SA (1995). An antidiabetic thiazolidinedione is a high affinity ligand for peroxisome proliferator-activated receptor γ (PPARγ). J Biol Chem.

[11] Nachman MW (2001). Single nucleotide polymorphisms and recombination rate in humans. Trends Genet.

[12] Lee JE, Choi JH, Lee JH, Lee MG (2005). Gene SNPs and mutations in clinical genetic testing: haplotype-based testing and analysis. Mutat Res.

[13] Rajasekaran R, Doss GP, Sudandiradoss C, Ramanathan K, Rituraj P, Sethumadhavan R (2008). Computational and structural investigation of deleterious functional SNPs in breast cancer BRCA2 gene. Sheng Wu Gong Cheng XueBao..

[14] Kamatani N, Sekine A, Kitamoto T, Iida A, Saito S, Kogame A (2004). Large-scale single-nucleotide polymorphism (SNP) and haplotype analyses, using dense SNP Maps, of 199 drug-related genes in 752 subjects: the analysis of the association between uncommon SNPs within haplotype blocks and the haplotypes constructed with haplotype-tagging SNPs. Am J Hum Genet.

[15] Krawczak M, Ball EV, Fenton I, Stenson PD, Abeysinghe S, Thomas N (2000). Human gene mutation database—a biomedical information and research resource. Hum Mutat.

[16] Prokunina L, Alarcón-Riquelme ME (2004). Regulatory SNPs in complex diseases: their identification and functional validation. Expert Rev Mol Med.

[17] Stenson PD, Mort M, Ball EV, Howells K, Phillips AD, Thomas NS (2009). The human gene mutation database: 2008 update. Genome Med.

[18] Alwi ZB (2005). The use of SNPs in pharmacogenomics studies. Malays J Med Sci..

[19] Abdelhamid FAA, Elhamid E, Mawada T, Elmahdi A, Thoiba I, Mohammed A, Aaya S, Mogammed E, Alaa A, Mohamed H, Mohamed A, Ammar A, Marwan B, Mohamed E, Mohamed H (2016). Computational analysis of single nucleotide polymorphism (Snps) in human MYC gene. J Bioinform Genomics Proteomics.

[20] Warde-Farley D, Donaldson SL, Comes O, Zuberi K, Badrawi R, Chao P (2010). The GeneMANIA prediction server: biological network integration for gene prioritization and predicting gene function. J Nucleic Acids Res..

[21] Ng PC, Henikoff S (2003). SIFT: predicting amino acid changes that affect protein function. Nucleic Acids Res.

[22] González-Pérez A, López-Bigas N (2011). Improving the assessment of the outcome of nonsynonymous SNPs with a consensus deleteriousness score condel. Am J Hum Genet..

[23] Venselaar H, TeBeek TA, Kuipers RK, Hekkelman ML, Vriend G (2010). Protein structure analysis of mutations causing inheritable diseases. An e-Science approach with life scientist friendly interfaces. BMC Bioinform.

[24] Bava KA, Gromiha MM, Uedaira H, Kitajima K, Sarai A (2004). ProTherm, version 4.0: thermodynamic database for proteins and mutants. Nucleic Acids Res.

[25] Capriotti E, Fariselli P, Calabrese R, Casadio R (2005). Predicting protein stability changes from sequences using support vector machines. Bioinformatic..

[26] Venselaar H, TeBeek TA, Kuipers RK, Hekkelman ML, Vriend G (2010). Protein structure analysis of mutations causing inheritable diseases. An e-Science approach with life scientist friendly interfaces. J BMC bioinform..

[27] Pettersen EF, Goddard TD, Huang CC, Couch GS, Greenblatt DM, Meng EC (2004). UCSF Chimera—a visualization system for exploratory research and analysis. J Comput Chem.

[28] Greene ME, Blumberg B, McBride OW, Yi HF, Kronquist K, Kwan K, Hsieh L, Greene G, Nimer SD (1995). Isolation of the human peroxisome proliferator activated receptor gamma cDNA: expression in hematopoietic cells and chromosomal mapping. Gene Expr.

[29] Meirhaeghe A, Amouyel P (2004). Impact of genetic variation of *PPAR* gamma in humans. Mol Genet Metab.

[30] Elbrecht A, Chen Y, Cullinan CA, Hayes N, Leibowitz M, Moller DE (1996). Molecular cloning, expression and characterization of human peroxisome proliferator activated receptors gamma 1 and gamma 2. Biochem Biophys Res Commun.

[31] Walczak R, Tontonoz P (2002). PPARadigms and PPARadoxes: expanding roles for PPARgamma in the control of lipid metabolism. J Lipid Res.

[32] Deeb SS, Fajas L, Nemoto M, Pihlajamaki J, Mykkanen L, Kuusisto J (1998). A Pro12Ala substitution in PPARgamma2 associated with decreased receptor activity, lower body mass index and improved insulin sensitivity. Nat Genet.

[33] Masugi J, Tamori Y, Mori H, Koike T, Kasuga M (2000). Inhibitory effect of a proline-to-alanine substitution at codon 12 of peroxisome proliferator-activated receptor-gamma 2 on thiazolidinedione-induced adipogenesis. Biochem Biophys Res Commun.

[34] Hasstedt SJ, Ren QF, Teng K, Elbein SC (2001). Effect of the peroxisome proliferator-activated receptor-gamma 2 pro(12)ala variant on obesity, glucose homeostasis, and blood pressure in members of familial type 2 diabetic kindreds. J Clin Endocrinol Metab..

